# Paper Collars

**Published:** 1869-03-15

**Authors:** 


### *Paper Collars.*

A correspondent wishes to be informed through THE JOURNAL whether the profession have noticed that the wearing of paper collars produces sore throat and enlargement of the tonsils? He says: "I find it almost entirely among men and boys who wear them. Is it not the arsenic or some other poison used in glazing them?" We have not the data from which to reply, although aware of a common opinion of this purport, which may, and may not, have some foundation in fact. *Quien sabe?*

